# 5.H. Round table: Health care systems, health service provision, and equity in health

**DOI:** 10.1093/eurpub/ckac129.309

**Published:** 2022-10-25

**Authors:** Organised by: EUPHA-HSR, EUPHA-EPI, Chair persons: Johan Hansen (EUPHA-HSR), Stefania Boccia (EUPHA-EPI)

## Abstract

The starting point for this round table is the observation that the research areas of health systems, health services and population health are usually seen as separate academic specialisations. This hampers the potential for getting insights into the role of health care systems and service provision in the development of population health and health inequalities, and of policies to reduce inequalities. As a result of the different mix of disciplines, with different approaches, different research and publication cultures, and different funding sources health systems research, health services research and population health research have tended to grow apart and to ignore the results from the other areas. In this round table session we will discuss the ways health care systems and health service provision influence inequalities in health. This implies looking at different levels of analysis. Health care systems and inequalities in population health refer to the macro level, and both are influenced by the same political and societal context. Health inequalities are also (and more strongly) influenced by structures and processes at macro level outside the health care system and service provision. Health service professionals form the intermediate level; their actual service delivery takes place at the micro level where health care professionals and users meet. Health care professionals and users of services bring their own attitudes, beliefs and resources that influence their interaction and consequent outcomes. Both meso and micro level are influenced by structures and institutions, in society in general as well as in the design of the health care system. The results of the interactions between health care professionals and users are (e.g.) decisions whether or not to use certain types of care, with consequences for the health and functional abilities of users. These decisions and their consequences are patterned by socio-economic characteristics of care users. These aggregate into patterns of inequality at the macro level. Over time, the influence of health care on population health has increased. The responsibility of health care for upstream causes of health inequalities can be strengthened through deliberate policies. With the (long-term) change of morbidity from infectious disease to chronic disease, prevention is often moving to programmes to support people in changing their lifestyle. This in itself exposes the relationships between health care and health inequalities, as those interventions that require a contribution from individuals tend to increase inequalities since those lacking resources will find it harder to participate. In this round table we present a proposal to integrate the three fields of research. We invite specialists from each of these fields and the audience of the round table to react to our proposal. The aim of this round table is to promote cross-disciplinary collaboration. The two organizing EUPHA sections cover the three areas of research in focus.

**Key messages:**

• The same political and societal context influences the health system, service provision and many of the social determinants of population health.

• The potential to address health inequalities through health care may have increased, and requires specific attention to integration of social care and different parts of health care.

**Speakers/Panellists:**

**Peter Groenewegen**

NIVEL, Utrecht, Netherlands

**Ilmo Keskimäki**

Finnish Institute for Health and Welfare, Helsinki, Finland

**Alastair Leyland**

University of Glasgow, Glasgow, UK

**Ellen Nolte**

LSHTM, London, UK

